# Thanksgiving

**Published:** 1892-12

**Authors:** 


### HALL'S JOURNAL OF HEALTH

TRUTH DEMANDS NO SACRIFICE; ERROR CAN MAKE NONE.

Vol. 39. DECEMBER, 1892. No. 12.

### THANKSGIVING.

The sweetest of all our holidays is the one set apart for thanksgiving and praise to the Great Giver of all Good for the bounty of the preceding harvest. Beginning with the New England states, whose several governors were wont to set apart the day by proclamation, the custom made its way to other states, until it embraced the whole family of states, thus becoming general and peculiar to this people. In earlier times it was not unfrequently the case, that the executives of bordering states appointed different days, with an interval of a week, for its observance, which enabled those of the same kin to interchange civilities at their hospitable gatherings, when the respective tables fairly groaned under the burden of the good things of this world. Eventually there was a demand for its recognition by the general government, and thus it became the custom for the Chief Executive to proclaim a day of thanksgiving and praise uniform with all the states, usually the last Thursday of November, and their respective governors fell into line and proclaimed the same day, until now, it is looked forward to as a settled thing.

It is customary, too, for the various church organizations to hold services at their houses of worship in the forenoon of Thanksgiving Day, leaving the afternoon free for enjoyment in a more congenial way, especially as to the younger members of families who make the occasion one of an annual re-union.

Fortunately the day has no sectarian significance. It is one on which those of all races and forms of belief can unite and kneel, at least metaphorically, at a common altar, in thankfulness for the fruits of the earth, and the blessings of a well ordered home.

Such a day has just been numbered with the past, but the sweet memories that gladdened many a heart will linger on through years of varied fortune, in the great world of selfishness and struggle to be met with on every hand, for such is the common lot.

